# Performance of Halo-Alkali-Tolerant Endophytic Bacteria on Hybrid *Pennisetum* and Bacterial Community under Varying Soil Conditions

**DOI:** 10.3390/microorganisms12061062

**Published:** 2024-05-24

**Authors:** Xia Li, Yiming Ding, Charles Obinwanne Okoye, Xiaoyan Geng, Huifang Jiang, Yongli Wang, Yanfang Wu, Lu Gao, Lei Fu, Jianxiong Jiang, Jianzhong Sun

**Affiliations:** 1 Biofuels Institute, School of Emergency Management, School of Environment and Safety Engineering, Jiangsu University, Zhenjiang 212013, China; 2222109050@stmail.ujs.edu.cn (Y.D.); charles.okoye@unn.edu.ng (C.O.O.); gengxiaoyan08@163.com (X.G.); 2112109002@stmail.ujs.edu.cn (H.J.); mdwyn@ujs.edu.cn (Y.W.); yanfang@ujs.edu.cn (Y.W.); lgao@ujs.edu.cn (L.G.); 18252585831@163.com (L.F.); jxjiang@ujs.edu.cn (J.J.); 2 School of Life Sciences, Jiangsu University, Zhenjiang 212013, China; 3 Department of Zoology & Environmental Biology, University of Nigeria, Nsukka 410001, Nigeria; 4 Library, Jiangsu University, Zhenjiang 212013, China

**Keywords:** halo-alkali-tolerance, endophytic bacteria, hybrid *Pennisetum*, salt stress, bacterial community

## Abstract

Halo-alkali soil threatens agriculture, reducing growth and crop yield worldwide. In this study, physicochemical and molecular techniques were employed to explore the potential of halo-alkali-tolerant endophytic bacteria strains *Sphingomonas* sp. pp01, *Bacillus* sp. pp02, *Pantoea* sp. pp04, and *Enterobacter* sp. pp06 to enhance the growth of hybrid *Pennisetum* under varying saline conditions. The strains exhibited tolerance to high salt concentrations, alkaline pH, and high temperatures. Under controlled conditions, all four strains showed significant growth-promoting effects on hybrid *Pennisetum* inoculated individually or in combination. However, the effects were significantly reduced in coastal saline soil. The best growth-promoting effect was achieved under greenhouse conditions, increasing shoot fresh and dry weights of hybrid *Pennisetum* by up to 457.7% and 374.7%, respectively, using irrigating trials. Metagenomic sequencing analysis revealed that the diversity and composition of rhizosphere microbiota underwent significant changes after inoculation with endophytic bacteria. Specifically, pp02 and co-inoculation significantly increased the *Dyella* and *Pseudomonas* population. Firmicutes, Mycobacteria, and Proteobacteria phyla were enriched in *Bacillus* PP02 samples. These may explain the best growth-promoting effects of pp02 and co-inoculation on hybrid *Pennisetum* under greenhouse conditions. Our findings reveal the performance of endophytic bacterial inoculants in enhancing beneficial microbiota, salt stress tolerance, and hybrid *Pennisetum* growth.

## 1. Introduction

Saline alkali soil is among the most devasting threats to agriculture at the global level, which has only emerged due to poor irrigation, continuous cropping of annual crops, and other agricultural methods [1]. It has been estimated that 20% of the world’s cultivated land (1.5 billion hectares) is affected by salt, leading to a significant reduction in soil productivity and crop yield, and up to 70% for some important cereal crops [2]. Considerable approaches have emerged to restore agricultural sustainability in saline soil to sustain the growing need for food supply, such as nanotechnology, agro-farming systems, and the use of tolerant bacteria [3]. Studies in recent decades have suggested the potential of endophytic bacteria as an eco-sustainable strategy to overcome this problem [4,5,6]. Endophytic bacteria are bacteria isolated from the plant endosphere, some of which can promote plant growth or stress tolerance and hold immense potential in developing eco-alternatives to pesticides and chemical fertilizers to reduce agricultural and environmental issues [4,7]. Numerous publications have reported the isolation of plant growth-promoting endophytic bacteria from rice, wheat, canola, maize, and tomato, some of which have been developed into commercial products [8,9,10,11,12,13]. Therefore, the exploitation of growth-promoting endophytic bacteria for plants such as hybrid *Pennisetum* could provide a novel solution to increase yield sustainably in saline soil.

Hybrid *Pennisetum* (*Pennisetum americanum* × *P. purpureum* Schumach) is a highly sterile interspecific perennial C4 bunchgrass, which shows the distinct advantages of high photosynthetic efficiency, high biomass yield, rich nutrient content, and strong resistance to environmental stressors. It is extensively cultivated in the tropics and subtropics of the world and is primarily used as animal feed [14,15]. Recently, with higher caloric value and cellulose content than most energy plants, hybrid *Pennisetum* has been widely applied as an energy plant in many countries to produce energy products, such as glucose, ethanol, methane, and biogas [16,17,18,19,20].

In our previous study, we isolated four strains of endophytic bacteria, *Sphingomonas* sp. pp01, *Bacillus* sp. pp02, *Pantoea* sp. pp04, and *Enterobacter* sp. pp06, which lessened the adverse impacts of salt stress and improved the growth of hybrid *Pennisetum* on under gnotobiotic condition, displaying great potential for developing microbial stimulant [21]. However, further research, including studying the morphological and physiological characteristics of these four strains to obtain the best fermentation conditions as the basis for large-scale production in the future is still needed to utilize them to develop microbial fertilizers. A significant universal problem is that although significant effects have been demonstrated by endophytic bacteria under controlled laboratory conditions, results often fail to reflect in the field, which hinders its broad use in agriculture [22,23,24]. Moreover, the inoculation method is also a critical factor in determining the performance of endophytic bacteria. For instance, when soybeans were inoculated with rhizobia, soil irrigation produced the highest nodule number and weight compared with the other three methods [25]. On the other hand, bacteria inoculated in soil performed better on plant biomass of Italian ryegrass than bacteria inoculated by seed soaking [26]. 

Based on the preceding, this study investigates the morphological and physiological characteristics of four endophytic bacteria and plant growth-promoting (PGP) effects of the four strains under greenhouse, gnotobiotic, and field conditions using two inoculation procedures. Additionally, the diversity of rhizosphere bacteria after inoculation with endophytic bacteria was analyzed to clarify the mechanism underlying their PGP effects.

## 2. Materials and Methods

### 2.1. Hybrid Pennisetum and Endophytic Bacteria

The seeds of hybrid *Pennisetum* (*Pennisetum americanum* × *P. purpureum* Schumach cv. Bangde No. 1) were purchased from Kaiyuan Grass Industry Co., Ltd. (Zhengzhou, Henan, China). Four endophytic bacterial strains, *Sphingomonas* sp. pp01, *Bacillus* sp. pp02, *Pantoea* sp. pp04, and *Enterobacter* sp. pp06 with accession numbers KM220524, KM886123, KP271021, and KP271022, were initially isolated from elephant grass elite No. 02 and maintained in our laboratory.

### 2.2. Bacteria Morphological Characteristics

Light and scanning electron microscopy (SEM) were applied to identify the colony and cellular morphologies of the four strains. Bacterial strains were cultured at 30 °C on Luria Bertani (LB) agar plates for colony morphological characterization. The colors and shapes of the colonies were observed under the microscope (Mshot MF53, Guangzhou, China) with 10× magnification. Photomicrographs were taken with the MS60 camera and MShot Image Analysis System V1 (MSHOT, Guangzhou, China). For SEM, bacterial cells were prepared as described before with some modifications [27]. Firstly, bacterial cells were fixed in 2.5% glutaraldehyde (*v*/*v*) overnight and dehydrated in a 30–100% graded ethanol series with each for 15 min. Subsequently, samples were immersed in 50% and then 100% isopentyl acetate with each for 1 h. After that, samples were freeze-dried, coated with gold palladium, and photographed with a SEM (JEOL JSM7001F, Tokyo, Japan).

### 2.3. Bacteria Physiological Characteristics

Bacterial growth was monitored by determining the OD_600_ of the bacterial suspension using a UV-visible spectrophotometer (Shimadzu UV2600, Kyoto, Japan). To determine growth curves, assays were carried out for 50 h in LB medium (pH 7.0) at 30 °C. Bacteria were grown for 45 h in an LB medium (pH 7.0) to determine optimum temperatures. Also, growth assays were carried out at 30 °C for 16 h to determine the optimum pH. In addition, the bacteria were grown in LB medium (pH 7.0) containing 0–11% sodium chloride at 30 °C for 16 h to determine the NaCl tolerance.

### 2.4. Gnotobiotic Trials

Pot experiments were set up using the previously described method to assess the PGP effects of the four strains on hybrid *Pennisetum* under gnotobiotic conditions [21]. The seeds of hybrid *Pennisetum* were surface sterilized and placed at 25 °C for germination. Seedlings were then sown into pots containing 40 g sterilized vermiculite, wetted with sterilized water, and grown at 25 °C under a long day photoperiod (16 h light/8 h dark). Bacterial suspension with a density of 10^8^ CFU/mL was harvested, re-suspended with sterile water, and poured into the vermiculite of the pots for inoculation of the seedlings by four individual strains singly or in combination of 4 strains with equal amounts. Control seedlings were treated with distilled water at equal volume. Ten pots were set up for each treatment. Four weeks later, the shoot length, shoot fresh weight (FW), and dry weight (DW) (dried in the oven continuously to constant weight) were measured.

### 2.5. Greenhouse and Field Trials

To further determine the PGP effects of the four bacterial strains, a greenhouse pot trial, and a field trial were conducted for hybrid *Pennisetum*. In the greenhouse experiment, soil was procured from Peilei Organic Fertilizer Co., Ltd. (Zhenjiang, Jiangsu, China) with the ammonium nitrogen (NH4^+^-N) of 92.48 ± 6.13 mg/kg, available potassium of 325.33 ± 7.02 mg/kg, available phosphorus of 19.91 ± 0.86 mg/kg, organic matter of 31.91 ± 14.29 mg/kg, organic carbon of 18.51 ± 8.29 mg/kg, and salt of 10.84 ± 0.98 mM/L (soil nutrient tester TPY 6A, Hangzhou, China). The bacterial suspension (10^5^–10^8^ CFU/mL) was prepared using the same method as in gnotobiotic trials. For seed soaking, hybrid *Pennisetum* seeds were soaked in bacterial suspension for one hour before sowing in plastic seedling-raising plates (105-well plates, 3.50 cm × 3.50 cm × 10.0 cm per well). The control seeds were soaked in the same volume of sterile water. For soil irrigating, hybrid *Pennisetum* seeds were sown in plastic seedling-raising plates, and 7 days later, the bacterial suspension (10^8^ CFU/mL) of four strains alone or in combination (pp01:pp02:pp04:pp06 = 1:1:1:1) was poured into the soil of seedling-raising plates. Control seedlings were soil irrigated with the same amount of sterile water. The shoot length, FW, and DW were measured four weeks later.

During the field trials, seedlings of 4 weeks inoculated with the bacteria by both seed soaking and soil irrigation were transplanted into the coastal saline soil in Yancheng City, Jiangsu Province, China (33°34.7′ N, 120°16.3′ E), and grown from early April to late October with the same method described in the greenhouse trials. The region has a subtropical monsoon climate, with an annual average temperature of 13.7–14.5 °C and an annual precipitation of 785.2–1309.5 mm. The chemical properties of the field soil (0–20 cm) were ammonium nitrogen (NH4^+^-N) of 15.22 ± 1.58 mg/kg, available potassium of 24.35 ± 4.32 mg/kg, available phosphorus of 22.59 ± 1.5 mg/kg, organic matter of 13.19 ± 3.66 mg/kg, organic carbon of 7.65 ± 2.12 mg/kg, salt of 45.59 ± 9.87 mM/L (TPY 6A Soil Nutrient Tester, Hangzhou, China). The plots were irrigated timely. No fertilization was applied to the soil. The experimental designs were randomized complete blocks with a row space of 60 cm. There were six treatments with three replications and 10 plants per replication.

### 2.6. DNA Extraction, Amplification, and 16SrRNA Sequence-Based Metagenomics

The microbial DNA extraction of the prepared soil samples (2 g for each sample) was carried out by centrifuging for 3 min at 10,000× *g*. Following the manufacturer’s instructions, genomic DNA extraction was carried out for each sample with the QIAGEN 47014 DNeasy^®^ PowerSoil^®^ DNA Kit (50) (Qiagen Inc., Germantown, MD, USA). Using the primer pairs 341F (5-CCT ACG GGN GGC WGC AG-3) and 805R (5-GAC TAC HVG GGT ATC TAA TCC-3), the bacterial 16S rDNA gene was amplified after genomic DNA was precisely quantified with a Qubit 3.0 Fluorometer (Life Technologies, Carlsbad, CA, USA). The PCR conditions include predenaturation at 94 °C for 3 min, 35 cycles of denaturation at 94 °C for 30 s, annealing at 55 °C for 30 s, extension at 72 °C for 30 s, and a final extension at 72 °C for 5 min. A Nanodrop One UV-Vis Spectrophotometer (Thermo Scientific, Wilmington, DE, USA) was used to assess the DNA quality. The Benagen Nanopore (Wuhan East Lake New Technology Development, Wuhan, China) analyzed high-throughput sequence. The operational taxonomic units (OTUs) were grouped using UPARSE (Version 7.1, https://drive5.com/uparse/, 28 April 2024) at a 97% level of sequence similarity. The taxonomy of the 16S rDNA gene sequences was assessed with the Ribosomal Database Project (Release 10, http://rdp.cme.msu.edu/, 28 April 2024). The alpha diversity, such as the ACE, Chao1, and Shannon index, was evaluated using Mouther (version 1.48.1 http://www.mothur.org/wiki/Classify.seqs, 28 April 2024). 

### 2.7. Statistical Analysis

One-way analysis of variance (ANOVA) was used to analyze the data with SPSS version 25.0 (IBM Corporation, Armonk, NY, USA). The Duncan Multiple Range Test (DMRT) was used to determine the mean separation at *p* < 0.05.

## 3. Results

### 3.1. Bacteria Morphological and Physiological Properties

**Figure 1:**
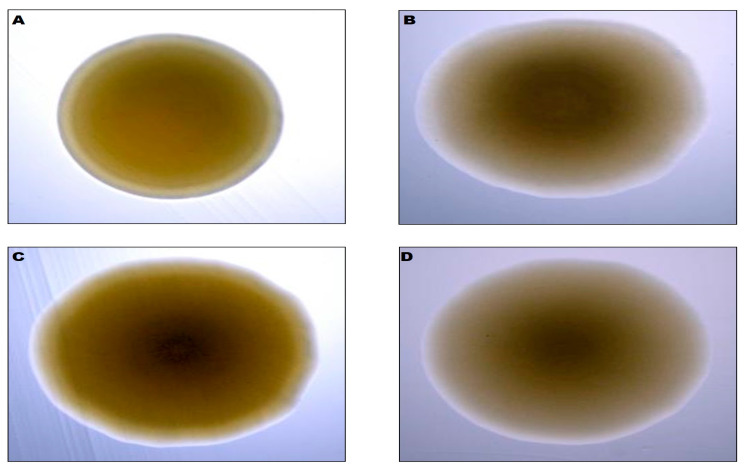
Typical colonies of four endophytic bacterial strains. Bacteria growing on LB medium 24 h after inoculation were observed under microscopy using a binocular with 10× magnification. (**A**) *Sphingomonas* sp. pp01; (**B**) *Bacillus* sp. pp02; (**C**) *Pantoea* sp. pp04; and (**D**) *Enterobacter* sp. pp06.

The colony morphologies of the four strains were observed after 24 h of culture on LB solid plates, which were different. *Sphingomonas* sp. pp01 formed smooth, yellow colonies (Figure 1A). The colony of *Pantoea* sp. pp02 was large, thick, round, white and opaque with a dry and wrinkled surface (Figure 1B). While the colony of *Pantoea* sp. pp04 was round, yellow, with neat edges (Figure 1C). The colony of *Enterobacter* sp. pp06 on LB plate was white, round and sticky (Figure 1D).

**Figure 2:**
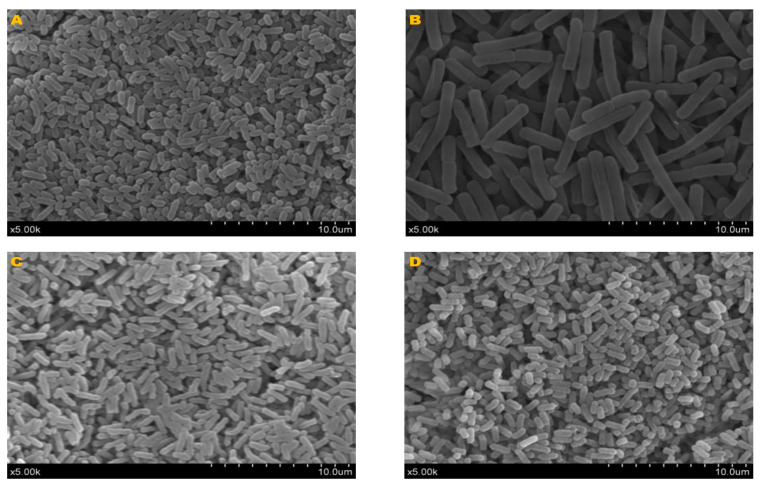
Images of endophytic bacterial cells. The bacterial cells were visualized by scanning electron microscopy after fixing and freeze-drying. Scale bars: 10.0 μm. (**A**) *Sphingomonas* sp. pp01; (**B**) *Bacillus* sp. pp02; (**C**) *Pantoea* sp. pp04; and (**D**) *Enterobacter* sp. pp06.

Morphological features of bacteria cells were visualized under SEM after pretreatment by fixing and freeze-drying. The morphologies are shown in Figure 2. *Sphingomonas* sp. pp01 was rod-shaped, 0.6–1.6 μm in diameter. *Bacillus* sp. pp02 was rod-shaped, round end, occurring singly, in pairs or chains, and 0.75–1 μm × 2.75–5 μm. *Pantoea* sp. pp04 was rod-shaped, 0.6–1.75 μm in diameter. *Enterobacter* sp. pp06 was rod-shaped, 0.68–1.5 μm in diameter.

**Figure 3:**
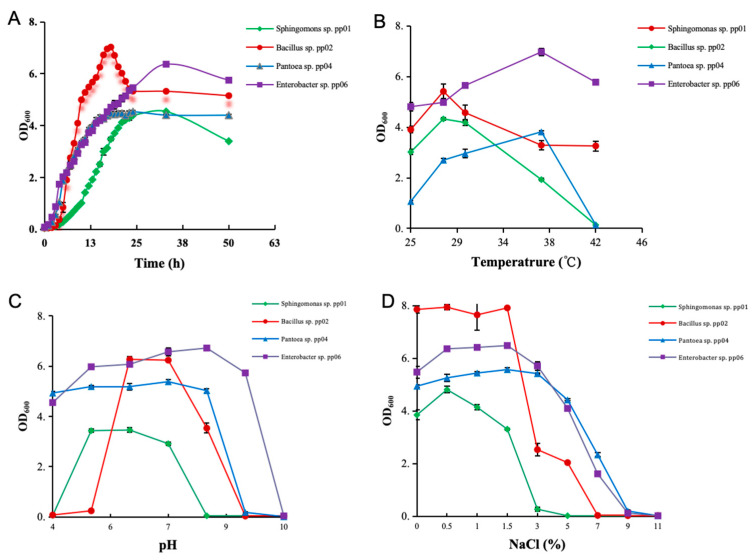
Growth curves and effects of temperature, pH, and NaCl concentrations on growth of the four strains. (**A**) Growth curves of the four strains in pH and temperature-controlled assays (temperature = 30 °C, pH = 7). (**B**) Effects of temperature on growth of the four strains in pH-controlled assays (pH = 7). (**C**) Effects of pH on growth of the four strains in temperature-controlled assays (temperature = 30 °C). (**D**) Effects of NaCl concentrations on growth curves of the four strains (temperature = 30 °C, pH = 7). Data represent the means from three independent experiments. Error bars indicate standard deviation (n = 3).

The growth performances of the four strains and their flexibility towards temperature, pH, and NaCl are shown in Figure 3. The growth curve of the four strains was plotted by measuring the OD_600_ absorbance of the bacterial solution under different culture times. As shown in Figure 3A, all strains reached the logarithmic period after 8 h of culturing in LB medium and peaked at about 40 h. The adaptation phase of pp01 was long and took about 9 h. The adaptation phases of the other three strains, pp02, pp04, and pp06, only took 3 h. The logarithmic phases of pp01, pp02, pp04, and pp06 occurred at 10–21 h, 3–10 h, 4–12 h, and 3–14 h, respectively, before entering the stationary phase. The decline phase occurred at 33 h, 19 h, 22 h, and 33 h for pp01, pp02, pp04, and pp06, respectively. 

Figure 3B showed their growth curves at different temperatures. Strains pp02 and pp06 can grow at all temperatures tested at 25–43 °C. At the same time, pp01 and pp04 grew well at temperatures from 25 °C to 37 °C, which can hardly grow when the temperature reaches 42 °C. Strains pp01 and pp02 exhibited the optimal growth rate at 28 °C, whereas the maximum growth rates of pp04 and pp06 were observed at 37 °C. Regarding pH, the four strains exhibited quite different ranges of tolerance (Figure 3C). Strain pp02 showed the narrowest pH tolerance between 5–9. The maximum growth was observed at pH 6. Strain PP01 can grow in the slight acid and neutral media at a pH value between 4 and 8, which grew optimally at pH 5, while pp04 and pp06 grow well with a wide pH tolerance ranging between 4 and 8 or 4 and 9, respectively, thus, showing maximum growth between pH 5 and 7 for both strains. 

Based on the turbidimetric readings of absorbance of four strains at different NaCl concentrations (0.5%, 1.0%, 1.5%, 3.0%, 5.0%, 7.0%, 9.0%, and 11.0%) for 16 h growth at 30 °C (Figure 3D), the different adaptation of these bacteria to NaCl concentrations were determined. Strain pp01 grew well under the NaCl concentrations of 0–1.5%. When the NaCl concentration exceeded 1.5%, its growth was inhibited and stopped at 3% NaCl concentration. Strain pp02 exhibited higher tolerance to NaCl than pp01 by showing slow growth at 3–5% but stopped at 7% NaCl. Strains pp04 and pp06 presented a wide tolerance range to NaCl concentration with no apparent inhibition of growth at 0–5% NaCl concentrations and could grow even under 7–9% NaCl. 

### 3.2. PGP Effects of Endophytic Bacteria under Gnotobiotic Conditions

**Figure 4:**
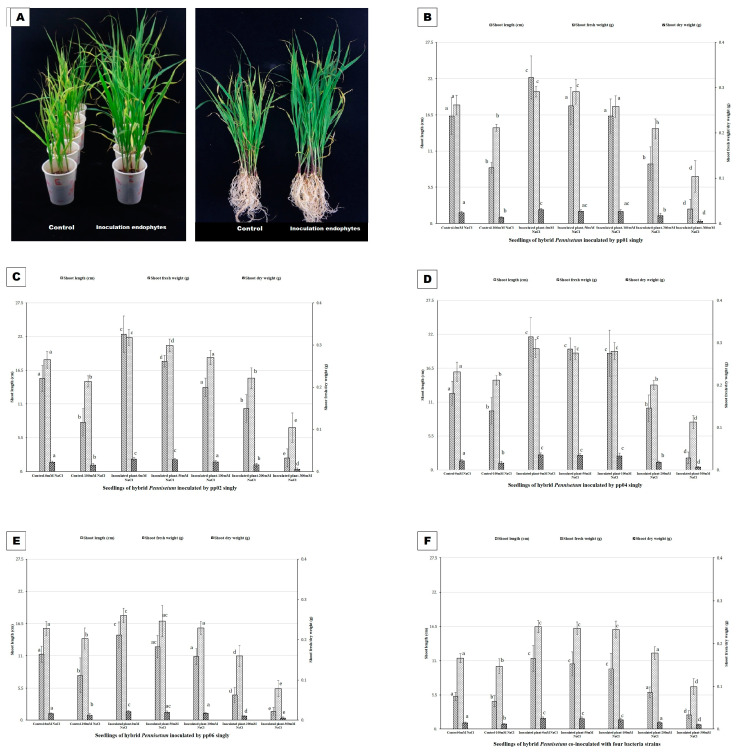
PGP effects of the four bacteria strains on hybrid *Pennisetum* under gnotobiotic condition. Seedlings of hybrid *Pennisetum* were inoculated with pp01, pp02, pp04, and pp06 singly or in combination with soil irrigation and then grew in sterilized vermiculite for 4 weeks. Bars denoted with the same letter for each compared parameter are not significantly different according to the Duncan Multiple Range Test (*p* = 0.05). Error bars indicate standard deviation (n = 10). (**A**) Hybrid *Pennisetum* seedlings under gnotobiotic condition. (**B**) Hybrid *Pennisetum* seedlings inoculated by pp01 singly. (**C**) Hybrid *Pennisetum* seedlings inoculated by pp02 singly. (**D**) Hybrid *Pennisetum* seedlings inoculated by pp04 singly. (**E**) Hybrid *Pennisetum* seedlings inoculated by pp06 singly. (**F**) Hybrid *Pennisetum* seedlings co-inoculated with four bacteria strains. Each group’s experiment was conducted independently with corresponding controls. Therefore, the comparison between (**B**) and (**F**) showed some differences.

Under gnotobiotic conditions, the PGP effects of the four strains varied under different salt stresses (0 mM NaCl~300 mM NaCl). They exhibited the best PGP effects under 0 mM NaCl, among which co-inoculation showed better effects than single inoculation by increasing the shoot length, shoot FW, and DW of hybrid *Pennisetum* to 4.38%, 116.20%, and 74.19%, respectively, compared with uninoculated plants (Figure 4). Under 50 and 100 mM NaCl, the PGP effects were weakened. With the increase in salt concentration, the growth rates of inoculated plants were diminished. However, the growth rates of inoculated plants showed no significant difference with or even better than uninoculated plants under 0 mM NaCl. Among them, co-inoculation and pp04 exhibited the best effects with 58.30%, 116.01%, and 81.72% increases in shoot length, shoot FW, and DW, respectively, compared to uninoculated plants under 100 mM NaCl. Under 200 mM NaCl, the growth of co-inoculation plants exhibited no difference from uninoculated plants under 0 mM NaCl. Even though the growth of seedlings inoculated with pp01, pp02, and pp04 were reduced under 0 mM NaCl, it was still better than the uninoculated plants under 100 mM NaCl, which implied that pp01, pp02, pp04 partially eliminated the detrimental effect of salt under 200 mM NaCl. Moreover, under 300 mM NaCl, neither co-inoculation nor single inoculations showed any PGP effects.

### 3.3. PGP Effects of Endophytic Bacteria under Coastal Saline Soil Conditions

**Figure 5:**
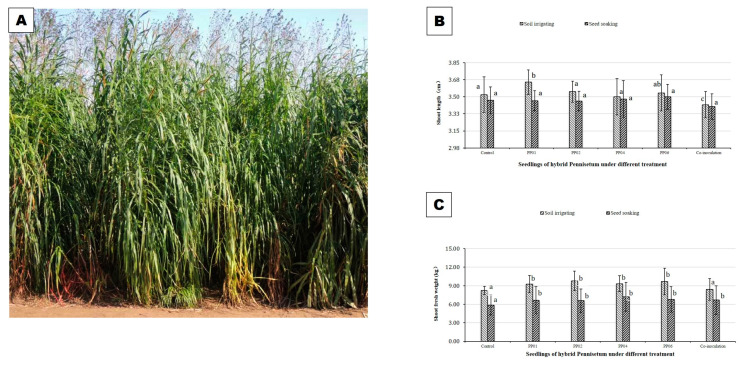
PGP effects of the four bacteria strains on hybrid *Pennisetum* under field conditions. Seedlings of hybrid *Pennisetum* were inoculated with pp01, pp02, pp04, and pp06 singly or in combination with soil irrigating or seed soaking. Then, 4-week seedlings were transplanted into the coastal saline soil (Yancheng City) and grew from early April to late October. There were six treatments with three replications and 10 plants per replication. (**A**) Hybrid *Pennisetum* seedlings under field condition; (**B**) shoot length; and (**C**) shoot fresh weight (FW). (Bars denoted with the same letter for each compared parameter are not significantly different according to the Duncan Multiple Range Test (*p* = 0.05). Error bars indicate standard deviation (n = 10).

Under field conditions of coastal saline soil (45.59 ± 9.87 mM/L salt content), though the PGP effects of the four strains were significantly reduced compared to gnotobiotic and greenhouse conditions, the growth of hybrid *Pennisetum* was still significantly promoted (Figure 5). Although there was no promotion effect on shoot length of hybrid *Pennisetum* under field conditions as gnotobiotic and greenhouse conditions, the inoculation of the four strains could eliminate the adverse effects of salt stress and caused the shoot FW to increase from 12.30% to 23.18% by both seed soaking or soil irrigating, except for co-inoculation by the soil irrigating method.

### 3.4. PGP Effects of Endophytic Bacteria under Greenhouse Conditions

**Figure 6:**
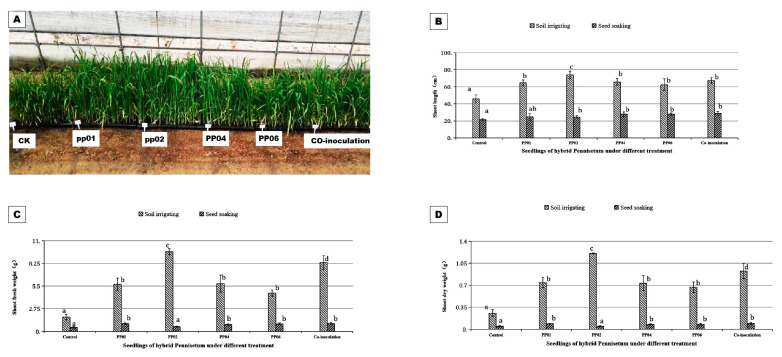
PGP effects of the four bacteria strains on hybrid *Pennisetum* under greenhouse conditions. Seedlings of hybrid *Pennisetum* were inoculated with pp01, pp02, pp04, and pp06 singly or in combination with soil irrigating or seed soaking and then grew in seedling-raising plates for 4 weeks. (**A**) Hybrid *Pennisetum* seedlings under greenhouse conditions; (**B**) shoot length; (**C**) shoot fresh weight (FW); and (**D**) shoot dry weight (DW). Bars denoted with the same letter for each compared parameter are not significantly different according to the Duncan Multiple Range Test (*p* = 0.05). Error bars indicate standard deviation (n = 10).

Under greenhouse conditions, the four endophytic bacteria exhibited better PGP effects than under gnotobiotic conditions (Figure 6). Strain pp02 demonstrated the best PGP effects in hybrid *Pennisetum* by soil irrigation, which increased the shoot length, shoot FW, and DW by 62.07%, 457.66%, and 374.68%, respectively, compared to that of the uninoculated plants. Co-inoculation, pp04, pp01, and pp06 exhibited slightly lower effects in the hybrid *Pennisetum* by increasing the shoot length, shoot FW, and DW to 47.8%, 381.7%, and 263.6%, respectively, with respect to uninoculated plants. However, when the seed soaking method was used, the PGP effects were greatly weakened. Co-inoculation, pp01, pp06, and pp04, exhibited similar PGP effects in the hybrid *Pennisetum*, which increased the shoot FW and DW to 94.6% and 79.8%, respectively, compared to uninoculated plants.

### 3.5. Bacterial Community Associated with Endophytic Bacteria under Greenhouse Conditions

#### 3.5.1. Diversity of Bacterial Community Associated with Endophytic Bacteria

**Figure 7:**
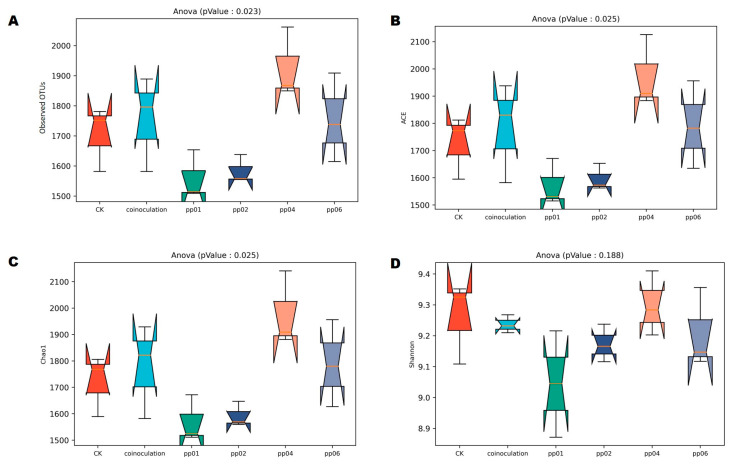
Alpha diversity indices based on 16S rDNA sequences. Box plots showing (**A**) observed OTUs, (**B**) ACE, (**C**) Chao1, and (**D**) Shannon indices of the bacterial community associated with endophytic bacteria in greenhouse soil samples. The significant difference was set at *p* < 0.05. Error bars indicate standard deviation (n = 3).

The microbial diversity of the rhizosphere was analyzed to determine the effects of endophytic bacteria under greenhouse conditions. The alpha diversity analysis in Figure 7 shows a significant difference in bacterial diversity among the endophytic bacteria inoculation and control groups. This indicates that inoculation of endophytic bacteria significantly affects bacterial diversity in the rhizosphere soil of hybrid Pennisetum.

**Figure 8:**
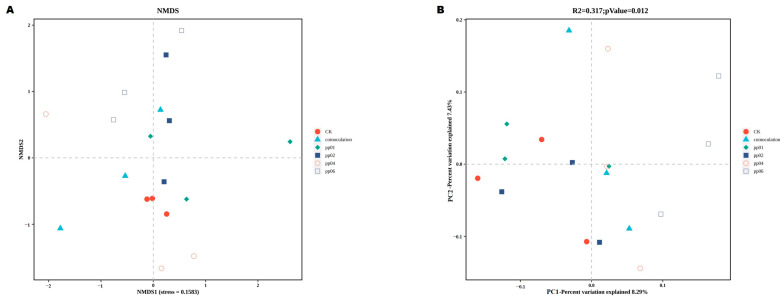
Bray–Curtis distance plots based on (**A**) non-metric multidimensional scaling (NMDS) with unweighted Unifrac dissimilarity and (**B**) principal coordinates analysis (PCoA) at the OTUs level of the bacterial community associated with the endophytic bacteria in greenhouse soil samples. Stress value: 0.158; ANOSIM value, *R*^2^: 0.317, and *p* = 0.012.

Among them, pp04 and co-inoculation significantly increased the bacterial species richness and diversity with OTUs (*p* = 0.023), ACE (*p* = 0.025), and Chao1 (*p* = 0.025) compared to the control group, except for the Shannon diversity index, which showed no significant difference (*p* = 0.188) between the samples. At the same time, pp02 and pp01 significantly decreased the bacterial diversity. Figure 8 shows the non-metric multidimensional scaling (NMDS) and principal coordinates analysis (PCoA) for the soil samples’ bacterial diversity at OTU levels based on the Bray–Curtis distance plots. The rank–order correlation between sample groups revealed a significant dissimilarity among the inoculated samples with a stress value of 0.158, with a pp02 and co-inoculation clustering evenly. At the same time, pp04, pp01, pp06, and CK were inconsistent across the ordination line (Figure 8A). The ANOSIM value of R^2^ = 0.317 revealed that the bacterial diversity in control and inoculated samples varies more at the OTUs level (*p* = 0.012), particularly between groups, where pp01, pp04, and pp06 clustered in PC2 (7.43%). Moreover, an apparent similarity was found among pp02 and co-inoculation, which clustered in PC1 (8.29%), showing a close similarity in bacterial diversity and richness (Figure 8B).

#### 3.5.2. Taxonomic Composition of the Bacterial Community Associated with Endophytic Bacteria

**Figure 9:**
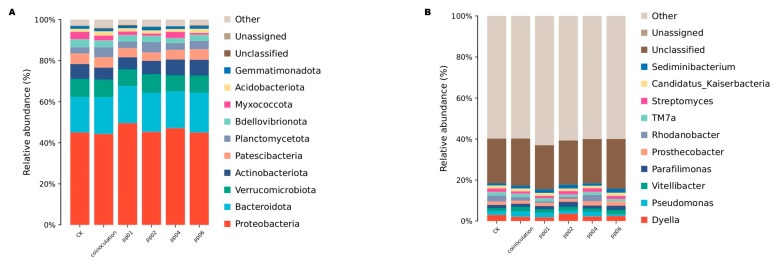
Relative abundances of the bacterial community associated with endophytic bacteria at the (**A**) phylum and (**B**) genus levels in the greenhouse soil samples.

The relative abundance of the top 10 bacteria phyla found in soil samples is shown in Figure 9A. These results constitute the average of three replications in both the inoculated and the control. Although there was no significant increase in the relative abundances of inoculated samples and the control, Bacteroidetes and Proteobacteria were the dominant phyla, accounting for 17.5–21.3% and 43.4–49.5, respectively. Besides, Gemratimonadota, Acidobacteriota, Myxooccota, Bdellovibrionota, Planctomycetota, Patescibacteria, Actinobacteriota, and Verrucomicrobiota represented the sub-dominant phyla (>1% of relative abundance) in the inoculated samples and the control, accounting for 1.42–1.83, 1.65–1.98, 0.80–2.45, 3.55–4.70, 4.43–6.50, 5.50–7.35, 6.55–8.24, and 8.50–11.25%, respectively.

Among the known 10 genera, *Sediminibacterium*, *Parafilimonas*, and *Vitellibacter* belong to the phylum Bacteroidetes; *Rhodanobacter*, *Pseudomonas*, and *Dyella* belong to the phylum Proteobacteria, while *Streptomyces*, *TM7a*, and *Prosthecobacter* belong to phyla Actinobacteriota, Patescibacteria, and Verrucomicrobiota, as well as the uncultured taxa, *Candidatus Kaiserbacteria.* However, the abundance of these genera did not differ (*p* > 0.05) across samples, except for pp02 and co-inoculation with significantly higher abundances of *Dyella* (*p* = 0.0490) and *Pseudomonas* (*p* = 0.0358), respectively (Figure 9B).

#### 3.5.3. Biomarkers of the Bacterial Community Associated with Endophytic Bacteria

**Figure 10:**
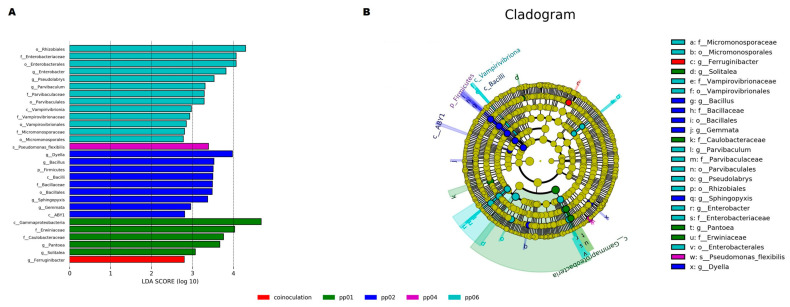
The differential abundance of bacterial taxa associated with endophytic bacteria in the greenhouse soil samples, based on LEfSe analysis. (**A**) Biomarkers are grouped according to their impact on various groups. When compared between samples, the biomarkers were significantly abundant (LDA > 4, *p <* 0.05). (**B**) The LEfSe-identified hierarchical taxonomic structure is displayed using a cladogram. A circle in the dataset represents a taxonomic unit, and colored circles or nodes indicate that the taxon represents a remarkably abundant group.

To further understand the effect of endophytic bacteria on the bacterial community, the abundance of bacteria community in greenhouse soil samples was analyzed (Figure 10). According to the linear discriminant analysis (LDA) effect size (LEfSe) analysis, 29 bacteria taxa were identified among the samples with significantly varied abundances (LDA > 4, *p* < 0.05) (Figure 10A). Comprehensively, pp06 was enriched with 13 bacteria taxa, followed by pp02 and pp01, enriched with 9 and 5 taxa, respectively, while pp04 and co-inoculation were enriched with only 1 taxon each. However, the abundant taxa were significantly enriched in pp02 with phyla Firmicutes, Planctomycetota, and Proteobacteria. These abundant taxa might be regarded as possible biomarkers (Figure 10B).

## 4. Discussion

In this study, the physiological characteristics of the four strains, such as growth curve, optimal temperature, pH value, and salt tolerance, provided suitable parameters that could favor their large-scale fermentation as promising microbial stimulants for hybrid *Pennisetum*. Meanwhile, the four strains were found to have high adaptability to salt and alkaline, regarded as halo-alkali-tolerant. Remarkably, both pp04 and pp06 are halo-alkali-tolerant and thermostable due to their ability to grow at 7–9% NaCl (*w*/*v*), pH of 8~9, and temperature of 42 °C. A similar study has reported that the salt content of saline-alkali land in the Dafeng City of Jiangsu Province is generally 0.15–0.45% (*w*/*v*), and the high range is more than 1% with the pH at 8.44 [28]. The pH of the plough layer in the coastal saline-alkali area of Cangzhou, China, is 7.84, and the salt content is 0.195% [29]. Thus, pp04 and pp06 can grow in most coastal saline-alkali lands in China. Since the use of bacteria has attracted more and more attention as a promising approach for remediating saline-alkali soil recently [30,31], pp04 and pp06 exhibited immense potential for further application in coastal saline-alkali soil remediation as bacteria resources.

The endophytic bacteria strains used in this study exhibited their abilities to remediate the detrimental impacts of salt stress and promote plant growth of hybrid *Pennisetum* under varying conditions, whether inoculated singly or in combination, which further confirmed their great potential for development as bio-stimulants. Among them, co-inoculation and pp02 showed the best effects. Co-inoculation showed the best PGP effects on hybrid *Pennisetum* under gnotobiotic and greenhouse conditions by seed soaking, which increased the shoot FW by 94.6% to 116.2%. Under greenhouse conditions with the soil irrigating method, co-inoculation also showed dramatic, second-best PGP effects, which increased the shoot FW and shoot DW to 381.7% and 263.6%. This result is consistent with prior studies showing that symbiosis with another beneficial microorganism may ameliorate the PGP effects of endophytic bacteria, which suggested that the combined application could be a promising strategy. For example, combined inoculation of five diazotrophic strains increased the sugarcane yield to 22.3~38.0 Mg ha^−1^ [32]. Another study reported that inoculation of *Daucus carota* L. combined with four bacteria strains enhanced soil fertility and plant growth [33].

Moreover, *Bacillus* sp. pp02 showed the best PGP effects under greenhouse conditions by soil irrigation, which increased the shoot FW and shoot DW to 457.7% and 374.7%. This result further verified the positive role of *Bacillus* sp. in the plant, which is a well-known and widely studied bacteria that alleviates salt stress and boosts plant growth, including *B. amyloliquifaciens*, *B*. *licheniformis*, *Bacillus siamensis*, *Bacillus velezensis*, etc. [34,35,36]. Thus, co-inoculation and pp02 presented more significant potential in developing microbial stimulants for hybrid *Pennisetum* in the future. 

However, though all four strains demonstrated positive effects on salt stress mitigation and the growth of hybrid *Pennisetum*, it is apparent that the effects varied significantly under different conditions. They showed the best effects in hybrid *Pennisetum* under greenhouse conditions by increasing the shoot FW to 457.66% (salt content, 10.84 ± 0.98 mM/L) followed by gnotobiotic conditions with an increase in shoot FW to 116.01% (50 mM NaCl). Under field conditions (salt content 45.59 ± 9.87 mM/L), the PGP effects dramatically decreased, increasing the shoot FW at 12.3~23.18%. Moreover, the four strains enhanced the shoot length of hybrid *Pennisetum* significantly by 15.7% to 62% under gnotobiotic and greenhouse conditions, but no effects were found under field conditions. These results lead to a similar conclusion to previous research. Some N2-fixing or P-solubilizing endophytic bacteria showed promising plant growth responses on sugarcane under laboratory or greenhouse conditions, but inconsistent beneficial effects were observed in field trials [37]. Endophytes that can promote the growth of turfgrass in low nutrients in greenhouse trials were found to have no effects in the field [38]. However, the reasons leading to inconsistent PGP bacteria results between controlled laboratory and field conditions are still unclear. According to Martínez-Viveros et al. [39], many experiments have shown that greenhouse conditions stimulate plant crop growth, leading to improved yield parameters and control of soil-borne pathogens. Meanwhile, replicating these successful PGP application results in field conditions has been challenging due to limited understanding of their ecology, survival, and activity in the plant rhizosphere. Therefore, in the following research, we will try to reveal the complex mechanism underlying the inconsistent PGP effects of the four strains in hybrid *Pennisetum* under laboratory and field conditions to promote their development as effective bio-stimulants.

The results also confirmed the critical role of inoculation procedures on the success of microbe inoculants. Under greenhouse conditions, when endophytic bacteria were applied by soil irrigation, co-inoculation exhibited the best PGP effects, followed by pp01, pp06, and pp04. But when endophytic bacteria were applied by seed soaking, pp02 exhibited the best PGP effect, followed by pp04, pp01, and pp06. Overall, soil irrigating treatment showed better PGP effects than seed soaking under greenhouse conditions, which led to a significant increase (to 167.5~457.7%, 162.2~374.7%) of shoot FW and DW of hybrid *Pennisetum* than uninoculated plants. While endophytic bacteria were applied by seed soaking, the increase (69~94.6%, 54.1~79.8%) of shoot FW and DW of hybrid *Pennisetum* was significantly lower. Under field conditions, although soil irrigating and seed soaking treatment exhibited a similar increase in shoot FW of hybrid *Pennisetum* compared to uninoculated plants, soil irrigating treatment exhibited higher shoot FW (to 8.4~9.8 kg) than that of seed soaking (only 6.6~7.23 kg). This result was consistent with previous reports. It was found that bacteria strains inoculated by soil irrigation performed better in plant biomass production than seed soaking [40]. However, other studies showed that seed soaking performed better than soil irrigating [41,42]. Reasons such as different inoculation methods causing different PGP effects may be attributed to plant morphological characteristics or the exudates released during plant growth stages, which could affect the development and persistence of bacteria in the rhizosphere [26,43]. Thus, soil irrigation was determined as the ideal inoculation method for the four strains in this study, but the reasons remain unknown.

The metagenomic sequencing analysis elucidated the effects of four endophytic bacteria strains on bacterial community in the rhizosphere of hybrid *Pennisetum* under greenhouse conditions. The inoculation of endophytic bacteria significantly affected the bacterial diversity and richness, with pp04 and co-inoculation leading to an increase, while pp02 and pp01 caused a decrease. The inoculation with *Penicillium oxalicum* P66 and *Aspergillus niger* P39 increased bacterial communities in the rhizospheres of soybean (Glycine max Merr. ‘Heinong 35’) and maize (*Zea mays* L. ‘Haiyu 6’) [44]. The inoculation of *Azospirillum* sp. B510 significantly increased the bacterial diversity in the rice rhizosphere [45]. At the same time, Gadhave et al. [46] suggested the application of *Bacillus* showed a significant reduction in the diversity and abundance of the native bacterial community. The application of PGPB as biocontrol or biofertilizer is often temporary and targeted at distinct bacterial taxa. An explanation for this is that after inoculating plants with PGP bacteria, the disturbance to the rhizosphere bacterial community may be complex, and the effects vary based on various factors, including the composition of inoculum, nutrient availability in soil, host plant species, and plant response to inoculum [47]. PCoA and NDMS analyses at the OTUs level showed that pp02 clustered with the co-inoculation sample clustered in PC1 (8.29%), showing a close similarity in bacterial composition and abundance. This partially explains the best growth-promoting effects of PP02 and co-inoculation on hybrid *Pennisetum* under greenhouse conditions.

In addition, the relative abundance of the top 10 bacterial phyla found in soil samples showed no overall change between the inoculated and control plants. Proteobacteria and Bacteroidetes were the dominant phyla (greater than 10% of relative abundance). Studies from China and Japan have shown a higher abundance of the two genera in saline soil samples inoculated with endophytic bacteria such as *Sphingomonas* sp. and *Bacillus* sp. [46,48,49,50]. However, the inoculation can still enrich some beneficial bacteria in the rhizosphere. *Dyella* was significantly higher in the pp02 sample at the genus level. *Dyella* has been identified with the potential to regulate plant growth. For instance, *Dyella* was found to be correlated positively with nodule number, nodule biomass, and nitrogenase activity in soybean plants [51]. *Dyella* also showed PGP activities in *Phaseolus vulgaris* and *Lespedeza* sp. [52]. Liu et al. [53] indicated that *Dyella* could improve mustard’s growth under greenhouse conditions; the present study showed that *Dyella* abundance might be linked to the best PGP effects on hybrid *Pennisetum* under greenhouse conditions through a positive association with pp02. *Pseudomonas* is among the major PGP halotolerant bacteria, which positively affected plant growth for their ability to supply phosphorus to plants, act as phytohormones to stimulate plant development, chelate and absorb iron through siderophores, and reduce the intermediate to ethylene (plant stress hormone) [54,55,56,57]. This can further be synchronized with the current study, where the co-inoculated sample showed an increased abundance of *Pseudomonas*, explaining the significant effects of promoting plant growth in hybrid *Pennisetum*. Moreover, the ability of *Dyella* and *Pseudomonas* to suppress plant pathogens has also been documented, which could limit the adverse effects of non-beneficial bacteria in pp02 and co-inoculation samples in the present study [58].

Furthermore, twenty-nine taxa with significantly differing abundances were discovered among the endophytic bacteria and their co-inoculation, according to the LEfSe analysis (Figure 10). Numerous studies have used the LEfSe to identify biomarkers between various sample groups linked to halophytes with disparate abundances [59,60]. Beneficial bacterial taxa belonging to three phyla, Firmicutes, Planctomycetota, and Proteobacteria, showed remarkable distribution and enrichment patterns in the *Bacillus* sp. pp02 sample. However, the inoculated samples were abundantly enriched with different taxa. The abundance of taxa other than the inoculated microbial taxa in analyzed metagenomics samples is still sketchy [35]. These more abundant bacteria in specific samples could represent enriched taxa with significant roles that alter the composition of communities [61]. According to the study of Dong et al. [62], the correlation of *Bacillus* sp. with Proteobacteria and Firmicutes may enhance the disease resistance of plants, promote bud growth, and increase overall crop yield since the nitrogen-fixing bacteria become enriched during the mature stages, distributing vital nutrients for the development of transformed axillary buds. This could circumspectly infer that *Bacillus* sp. pp02 actively reshapes the beneficial microbiota through positive interaction to enhance adaptability during environmental stress to promote plant growth under greenhouse conditions. As an important member of the soil microbial community, fungi play an important role in soil nutrient cycling, improving plant growth and health [63,64,65]. Thus, the consideration of both fungi and bacteria together can better analyze the impact of endophytic bacteria on rhizosphere soil. Therefore, in the future, we can conduct specific research on soil fungal diversity in order to provide a more in-depth and comprehensive explanation of the impact of endophytic bacteria on the soil microbial community of hybrid *Pennisetum*.

## 5. Conclusions

The four strains displayed remarkable adaptability to salt and alkaline conditions, making them valuable candidates for potential microbial stimulants for hybrid *Pennisetum* cultivation. Co-inoculation and *Bacillus* sp. pp02 emerged as the most promising strains for enhancing the growth of hybrid *Pennisetum*. Their ability to alleviate salt stress and promote plant growth was particularly evident under controlled greenhouse conditions. The performance of these strains varied under different environmental conditions, especially in greenhouse conditions, highlighting the complexity of translating laboratory results into practical applications. The choice of inoculation method also played a vital role in determining the performance of the bacterial inoculants. Soil irrigation appeared to be the most effective method, emphasizing the importance of rhizosphere interactions in promoting plant growth under greenhouse conditions. Metagenomic sequencing analysis provided valuable insights into the potential of *Bacillus* sp. pp02 samples to interact with beneficial bacterial phyla, including Bacteroidetes, Proteobacteria, and Firmicutes, in improving plant growth under greenhouse conditions. Furthermore, the study showed that inoculating endophytic bacteria altered the community composition and richness of bacteria in the rhizosphere soil of hybrid *Pennisetum* since pp02 and co-inoculation were enriched with Dyella and Pseudomonas abundance, respectively, thus suggesting the necessity for further studies, such as tracking the fate of inoculated strains over time to better understand why the inoculated samples were enriched with different genera. This may be helpful to investigate the specific environmental conditions, complex mechanisms underlying their interactions, and sampling procedures.

## Data Availability

The metagenome sequencing data used in this study was submitted to the National Center for Biotechnology Information database (https://www.ncbi.nlm.nih.gov/) under the BioProject 1D: PRJNA1062135.
